# Cryptococcal Cell Morphology Affects Host Cell Interactions and Pathogenicity

**DOI:** 10.1371/journal.ppat.1000953

**Published:** 2010-06-17

**Authors:** Laura H. Okagaki, Anna K. Strain, Judith N. Nielsen, Caroline Charlier, Nicholas J. Baltes, Fabrice Chrétien, Joseph Heitman, Françoise Dromer, Kirsten Nielsen

**Affiliations:** 1 Department of Microbiology, Medical School, University of Minnesota, Minneapolis, Minnesota, United States of America; 2 Department of Pathology and Laboratory Medicine, School of Medicine, University of North Carolina at Chapel Hill, Chapel Hill, North Carolina, United States of America; 3 Institut Pasteur, Unité de Mycologie Moléculaire and CNRS URA3012, Paris, France; 4 Faculté de médecine; Université Paris XII; APHP Hôpital Henri Mondor and INSERM U955 team10, Paris, France; 5 Departments of Molecular Genetics and Microbiology, Medicine, and Pharmacology and Cancer Biology, Duke University Medical Center, Durham, North Carolina, United States of America; Carnegie Mellon University, United States of America

## Abstract

*Cryptococcus neoformans* is a common life-threatening human fungal pathogen. The size of cryptococcal cells is typically 5 to 10 µm. Cell enlargement was observed *in vivo*, producing cells up to 100 µm. These morphological changes in cell size affected pathogenicity via reducing phagocytosis by host mononuclear cells, increasing resistance to oxidative and nitrosative stress, and correlated with reduced penetration of the central nervous system. Cell enlargement was stimulated by coinfection with strains of opposite mating type, and *ste3* ***a*** *Δ* pheromone receptor mutant strains had reduced cell enlargement. Finally, analysis of DNA content in this novel cell type revealed that these enlarged cells were polyploid, uninucleate, and produced daughter cells *in vivo*. These results describe a novel mechanism by which *C. neoformans* evades host phagocytosis to allow survival of a subset of the population at early stages of infection. Thus, morphological changes play unique and specialized roles during infection.

## Author Summary

*Cryptococcus neoformans* is a common life-threatening opportunistic human fungal pathogen. *C. neoformans* grows as a budding yeast *in vitro* with typical cell sizes ranging from 5 to 10 µm in diameter. Early reports suggested the presence of enlarged cells in human infections yet the identity of these cells and their role in virulence remained uncharacterized. Changes in cellular morphology are also observed in the mouse inhalation model of cryptococcosis. These enlarged “titan” cells accounted for 20% of the cells in the lungs. Titan cell formation was found to be regulated by a G-protein coupled receptor (GPCR) signal transduction pathway associated with pheromone sensing. Analysis of titan cells revealed uninucleate, polyploid cells that reproduced by budding, suggesting alterations in the regulation of cell growth and mitosis. Titan cell formation also affected host-pathogen interactions by reducing phagocytosis by host mononuclear cells and was correlated with reduced dissemination within the host. These results describe a novel mechanism by which *C. neoformans* undergoes morphogenesis to evade host phagocytosis, leading to the hypothesis that titan cell formation allows survival of a subset of the population and plays a unique and specialized role during infection.

## Introduction

Unicellular organisms exhibit morphological changes under a wide variety of environmental conditions. In many pathogenic fungi, the ability to switch cell morphology is integral to the infection cycle. Dimorphic fungi, such as *Blastomyces dermatitidis* and *Histoplasma capsulatum*, grow in the environment in a hyphal form. When a susceptible host inhales spores, these fungi grow as yeasts. This change in morphology is induced by the high mammalian body temperature [1], [2], [3]. Other pathogenic fungi, such as *Candida albicans* and *Coccidioides immitis*, change to specific cell morphologies based on environmental cues or stage of infection [4], [5], [6]. Morphological changes in the pathogenic fungus *C. albicans* affect tissue tropism and dissemination. Hyphal cells are important in the invasion of host tissues, while yeast cells can easily disseminate through the blood and lymph systems to spread the infection [5], [7]. Additionally, phagocytosis of yeast cells induces differentiation into hyphal cells [6].

*Cryptococcus neoformans* is an opportunistic fungal pathogen that is most commonly associated with disease in immunocompromised patient populations, such as HIV/AIDS patients, transplant recipients, patients with lymphoid disorders, chronic treatment with corticosteroids, or patients undergoing certain types of chemotherapies [8], [9], [10]. *C. neoformans* presents clinically as skin lesions, pneumonia, or meningitis [11]. Over 30% of the HIV/AIDS population in Sub-Saharan Africa present with cryptococcal meningitis and cryptococcosis is currently the fifth leading cause of fatalities in this region [12].

Infection with *C. neoformans* begins when desiccated yeast cells or spores are inhaled and lodge in the alveoli of the lungs. Cryptococcosis occurs when yeast cells disseminate to the bloodstream and ultimately penetrate the blood-brain barrier (BBB) [10], [13]. While the exact mechanism for trafficking from the lungs to the central nervous system (CNS) remains unknown, interactions with host phagocytes and the endothelial cells of the BBB have been shown to be important in this process [14], [15], [16], [17], [18], [19], [20], [21], [22].

Morphogenesis in *C. neoformans* has primarily been observed as a result of pheromone signaling and mating [23], [24]. There are two varieties of *C. neoformans*: *neoformans* and *grubii*. Historically, mating has been studied *in vitro* in var. *neoformans* even though the vast majority of human cryptococcosis cases are caused by var. *grubii*.

*C. neoformans* has two mating types: **a** and α. Mating is initiated when pheromone (**a** or α) secreted by one mating type binds to the pheromone receptor, Ste3α or Ste3**a** respectively, of the other mating type to trigger a mitogen-activated protein kinase (MAPK) signaling cascade [23], [25]. Pheromone signaling results in morphological changes in var. *neoformans*, including germ tube formation by mating type α cells and enlargement of mating type **a** cells [23], [24]. Pheromone-induced MAPK signaling ultimately results in fusion of **a** and α cells followed by dikaryotic filamentation. Dikaryotic hyphae eventually give rise to basidia where nuclear fusion occurs and meiosis produces haploid spores [23], [26]. In var. *grubii*, no *in vitro* morphogenesis in wild-type strains has been observed during early pheromone signaling, although hyphal formation and basidium production mimic that seen in var. *neoforman*s [27].

In this study, we show that cell enlargement is observed *in vivo* in var. *grubii*, and that this cell enlargement can be regulated by pheromone signaling. Additionally, we show that these morphological changes in cell size affect pathogenicity by altering phagocytosis and dissemination to the central nervous system (CNS). Finally, we characterized DNA content of this novel cell type to reveal that these enlarged cells are polyploid.

## Results

### Morphological changes in *C. neoformans* var. *grubii* cells *in vivo*

**Figure 1:**
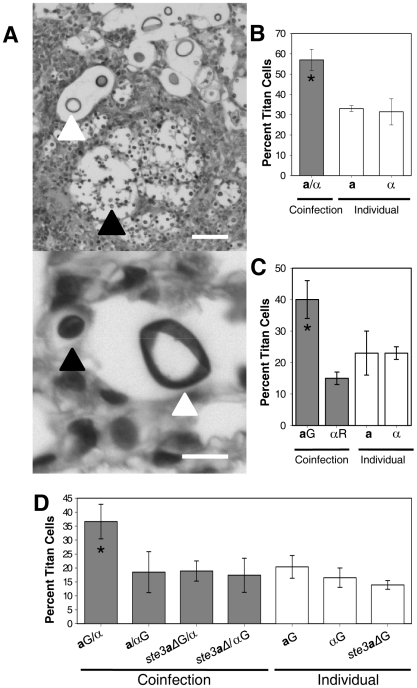
Titan cells in the lungs of coinfected mice. **A**) Mice were coinfected with an approximate 1∶1 ratio of **a**:α intranasally at a final concentration of 5×10^4^ cells. Lung sections were stained with periodic acid Schiff (PAS) 14 days (top) or 3 days (bottom) post-infection. White arrow denotes *C. neoformans* cells >10 µm in diameter. Black arrow denotes cells ≤10 µm in diameter. Top: bar  = 100 µm, bottom: bar  = 10 µm. **B**) The number of small cells (≤10 µm) and titan cells (>10 µm) were quantified in single and coinfections at 7 days post-infection. >500 cells were counted per treatment per mouse. Error bars indicate SD from 3 mice per treatment. Asterisk indicates p<0.01 in pair-wise comparisons to all other samples. **C**) Mice were infected by inhalation with 5×10^4^ of either **a** cells labeled with AlexaFluor 488 (green, **a**G), α cells labeled with AlexaFluor 594 (red, αR), or coinfected with an approximate 1∶1 ratio of each mating type. At 2–3 days post-infection, animals were sacrificed and unstained tissue sections were examined for fluorescence and cell size. >80 cells were analyzed per mouse per treatment. Data are representative of three independent experiments with three mice per treatment. Error bars indicate SD. Asterisk indicates p<0.01 in pair-wise comparisons to all other samples. **D**) Mice were infected with 5×10^7^ cells by inhalation of either **a**, α, or *ste3* ***a*** *Δ* cells labeled with AlexaFluor 488 or coinfected with one labeled and one unlabeled strain. Cells obtained by bronchoalveolar lavage (BAL) were fixed and examined by microscopy for green fluorescence and cell size. >500 cells were examined per animal. Error bars indicate SD from four mice per treatment. Asterisk indicates p<0.01 in pair-wise comparisons to all other samples; p-values >0.2 were observed for other pair-wise comparisons.

Pheromone signaling in *C. neoformans* is known to cause morphological changes including formation of conjugation tubes, dikaryotic filaments, and production of basidia and spores [23], [24], [26], [28]. Mating type **a** cell enlargement has also been observed in confrontation assays [23]. Cell enlargement has been observed in both human and mouse specimens [29], [30], [31], [32]. Thus, we systematically analyzed cellular morphology in various tissues of mice intranasally infected with var. *grubii* mating type **a** or α strains or mice coinfected with both mating types to determine the effect of pheromone signaling and mating type on *in vivo* cell morphology. Histopathologic tissue sections from the lungs, heart, spleen, liver, kidneys, and brain at 1, 2, 3, 7, 14, and 21 days post-infection were examined for changes in cryptococcal cell morphology. Dramatic changes in cryptococcal cell size were observed in the lungs, although a few cells with increased cell size were also observed in the spleen and brain at late time points (**Figure 1A**, **Figure S1**). Most fungal cells in the lungs remained small (5–10 µm in diameter) resembling yeast cells grown in rich medium *in vitro*. However, a proportion of the cryptococcal cells in the lungs were much larger. For ease of reference, we designated this group of enlarged cryptococcal cells as “titan” cells. These titan cells were >10 µm in diameter, with some cell sizes approaching 50 to 100 µm in diameter (**Figure 1A**). Titan cell diameter measurements were based on actual cell body size and excluded capsule changes which were highly variable. Titan cells were observed as early as 1 day post-infection in the lungs, accounted for approximately 20% of the cryptococcal cells in the lungs by 3 days post-infection, and remained relatively constant throughout the rest of the infection (**Figure 1B, 1C**; **Figure S1**). Titan cells were occasionally observed in the spleen and brain but at low levels (**Figure S1**). In contrast, coinfection with both mating types resulted in an increase in titan cell production to almost 50% of the cells present in the lungs (**Figure 1B, 1C**).

**Figure 2:**
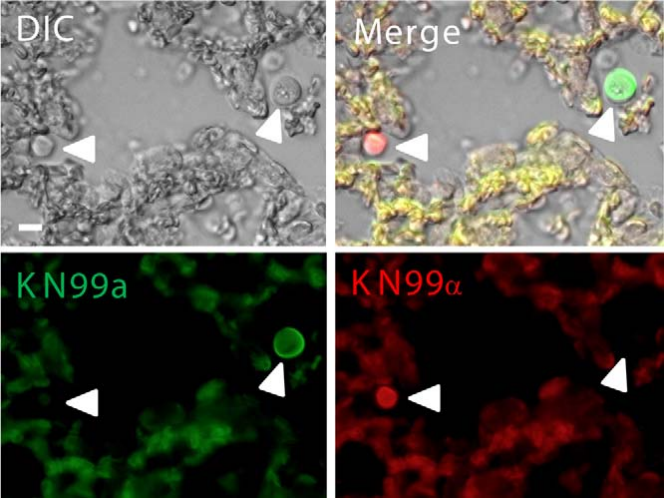
Fluorescently labeled a (green) and α (red) cells in the lungs during coinfection. *C. neoformans* **a** and α strains were combined with AlexaFluor 488 (green) or AlexaFluor 594 (red), respectively, and incubated for 20 minutes. Cells were washed with PBS to remove excess dye. Mice were inoculated with an approximate 1∶1 ratio of **a:**α cells at a final concentration of 5×10^7^ cells. At 2 days post-infection animals were sacrificed, lungs extracted, fixed in 10% buffered formalin, paraffin-embedded, and 5 µm sections generated. Host tissues are autofluorescent at both wavelengths resulting in a yellow color upon overlay. White arrows denote fluorescent *C. neoformans* cells. Bar  = 20 µm.

Because pheromone signaling induces mating type **a** cell enlargement during *in vitro* mating of var. *neoformans*, we hypothesized that the increase in titan cell formation during coinfection was specific to mating type **a** cells. To test this hypothesis, we differentially stained **a** cells with AlexaFluor 488 (green) and α cells with AlexaFluor 594 (red) prior to intranasal inoculation of mice. Mice were sacrificed at 1–3 days post-infection, and unstained histopathological sections were examined for cryptococcal cell fluorescence (**Figure 2**). At 1 day post-infection, no difference in the proportion of **a** or α titan cells in individual or coinfections was observed (data not shown). However, at 2–3 days post-infection, the proportion of mating type **a** titan cells in coinfections increased while the α titan cell proportion remained equivalent to the individual infections (**Figure 1C**). Almost half of the stained mating type **a** cells in coinfected lungs had converted to titan cells.

To further quantify titan cell formation during coinfection, cells were differentially stained green with AlexaFluor 488 prior to intranasal instillation with the following treatments: **a** only (green), α only (green), **a**(green)/α, or **a**/α(green). At 3 days post-infection, bronchoalveolar lavage (BAL) was performed. The resulting mix of cryptococcal and mouse cells was immediately fixed and the proportion of green titan cells was determined by microscopic examination (**Figure 1D**, see below). Similar to the tissue sections, approximately 20% titan cells were observed in the individual infections with no difference in titan cell formation between the two mating types (p = 0.2, **Figure 1D**). In the coinfections, mating type α titan cell formation remained at the basal level (p>0.64, **Figure 1D**) while mating type **a** titan cell formation increased (p<0.01, **Figure 1D**). In the BAL samples, titan cell formation was increased to 37% of the fluorescently labeled **a** cells. Additionally, the proportion of titan cells in the tissue sections and the BAL samples was similar, suggesting that BAL samples obtained at 3 days post-infection provide an accurate representation of the cryptococcal cells present in the lungs.

### Pheromone signaling affects titan cell formation

Cryptococcal cells can signal to cells of the opposite mating type using pheromones. Pheromone from one cell type binds to a G-protein coupled receptor, Ste3, on the opposite cell type to trigger a MAPK signaling cascade that can alter cell morphology [23], [25]. We examined whether the increase in titan cell formation during coinfection was due to pheromone signaling by mutating the *STE3* ***a*** pheromone receptor gene.

The *STE3* ***a*** coding sequence was replaced with a nourseothricin (NAT) resistance gene by homologous recombination. The resulting *ste3* ***a*** *Δ* mutant lacks the receptor to recognize α pheromone, thus the pheromone signaling pathway is not activated and *ste3* ***a*** *Δ* mutant cells fail to mate with α strains. Two independent congenic mutants were generated: *ste3* ***a*** *Δ*#1 and *ste3* ***a*** *Δ*#2. Both *ste3* ***a*** *Δ::NAT* mutant strains were sterile in mating assays with an α strain (data not shown). Murine survival assays verified that the *ste3* ***a*** *Δ* mutants had equivalent virulence to the parental strain KN99**a** and no differences in mean survival time were observed between wild-type and *ste3* ***a*** *Δ* coinfections (**Figure S2**).

To assess the role of pheromone sensing in titan cell formation, BAL samples were obtained from mice infected with fluorescently labeled *ste3* ***a*** *Δ*#1 only (green), *ste3* ***a*** *Δ*#1(green)/KN99α, or *ste3* ***a*** *Δ*#1/KN99α(green) (**Figure 1D**). Average titan cell formation was 14% in the *ste3* ***a*** *Δ*#1 infection, which was lower than the average for wild-type **a** cells (p = 0.03), but similar to wild type α cells (p = 0.22). In contrast, no increase in titan cell formation was observed in coinfections with the *ste3* ***a*** *Δ*#1 mutant (p>0.6, **Figure 1D**). Thus, the increase in titan cell formation by mating type **a** cells during coinfection requires the Ste3**a** receptor. However, the presence or absence of the Ste3**a** pheromone receptor has little effect on the basal level of titan cell formation observed in individual infections.

### Pheromone signaling alters dissemination to the central nervous system

To examine the role of titan cell formation in pathogenicity, individual and coinfections with the wild-type and *ste3* ***a*** *Δ* mutant strains were compared. The *Cryptococcus* infectious cycle can be divided into three stages: an initial pulmonary infection (lungs), dissemination (spleen), and penetration of the CNS (brain). Previous studies with *C. neoformans* var. *grubii* congenic strains showed no differences in virulence between the **a** and α mating types [26]. However, coinfection with both mating types simultaneously resulted in reduced **a** cell penetration of the CNS [33]. Interestingly, while **a** cell CNS penetration was reduced compared with α cells, both cell types had equivalent accumulation at the first two stages of infection.

**Figure 3:**
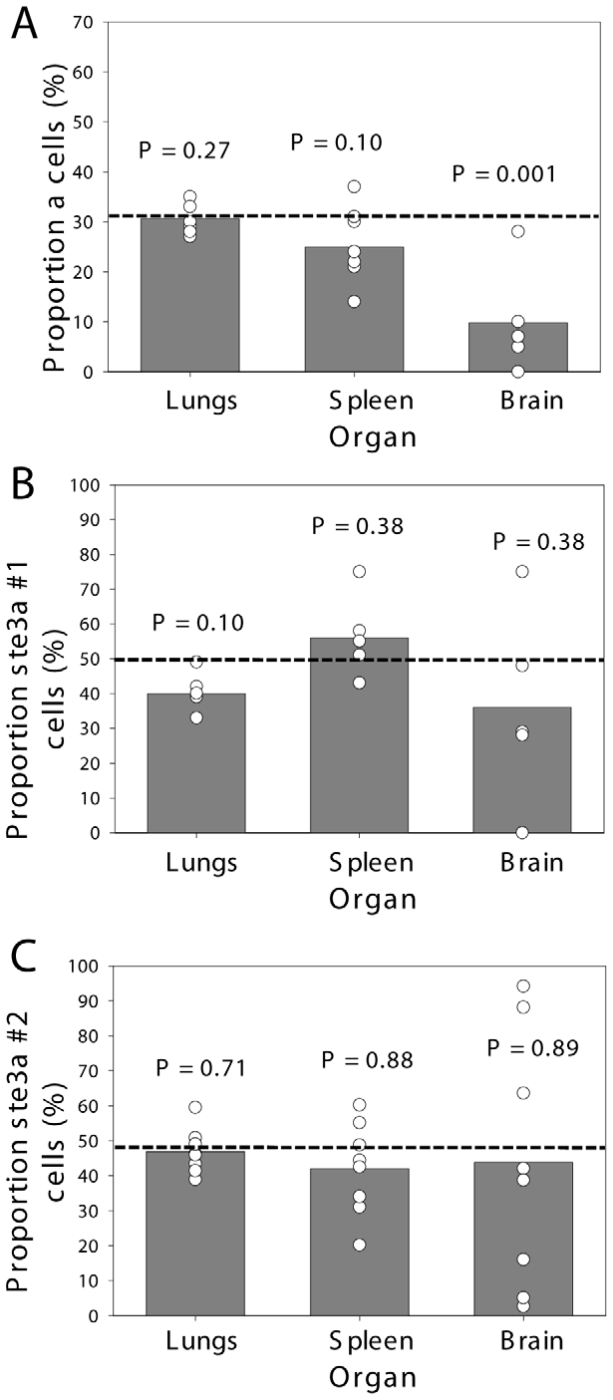
*C. neoformans* pheromone receptor mutant strains penetrate the CNS during coinfection. Mice were coinfected intranasally with an approximate 1∶1 ratio of **A**) **a:**αNAT, **B**) *ste3* ***a*** *Δ*#1:α, or **C**) *ste3* ***a*** *Δ*#2:α at a final concentration of 5×10^4^ cells. The actual proportion of **a** cells in the infecting inoculum was determined by growth on selective medium and is plotted as a horizontal dashed line. At 21 days post-infection animals were sacrificed, the lungs, brain, and spleen were homogenized and serial dilutions plated. >500 colonies per organ per mouse were isolated and assayed for drug resistance to determine mating type. The proportion of **a** cells is plotted with open circles denoting values from individual animals and bar height representing the geometric mean. To determine *P*-values, Wilcoxon rank sum analysis was performed on the measured number of **a** and α cells compared with the expected number, assuming that both strains remained at the initial inoculum proportions.

During coinfection, only mating type **a** cells displayed an increase in titan cell formation and a subsequent reduction in CNS penetration. Thus we hypothesized that pheromone signaling and the resulting increase in titan cell formation reduces **a** cell CNS penetration. To determine whether pheromone signaling affected dissemination to the brain during coinfection, we compared wild-type and *ste3* ***a*** *Δ* mutant strains for CNS penetration when coinfected with α (**Figure 3**). In both wild-type and *ste3* ***a*** *Δ* coinfections, the number of **a** and α cells recovered from the spleen and lungs was equivalent to the proportion of the two cell types in the initial inocula (p>0.1). These data show, even at late time points, alterations in titan cell production in response to pheromone signaling do not affect persistence of the cells in the lungs. However, a significant decrease was seen in the proportion of wild-type **a** cells recovered from the brain (p = 0.001, **Figure 3A**). In contrast, coinfections with the *ste3* ***a*** *Δ* mutants restored **a** cell accumulation in the CNS to levels equivalent to the initial inocula (p>0.4, **Figure 3B, C**). Both independent *ste3* ***a*** *Δ* mutants showed similar results. Together, these data suggest that pheromone signaling during **a**/α coinfection affects the pathogenicity of **a** cells by increasing titan cell formation which inhibits the ability of **a** cells to establish a CNS infection.

### Coinfection does not affect blood brain barrier penetration upon IV injection

**Figure 4:**
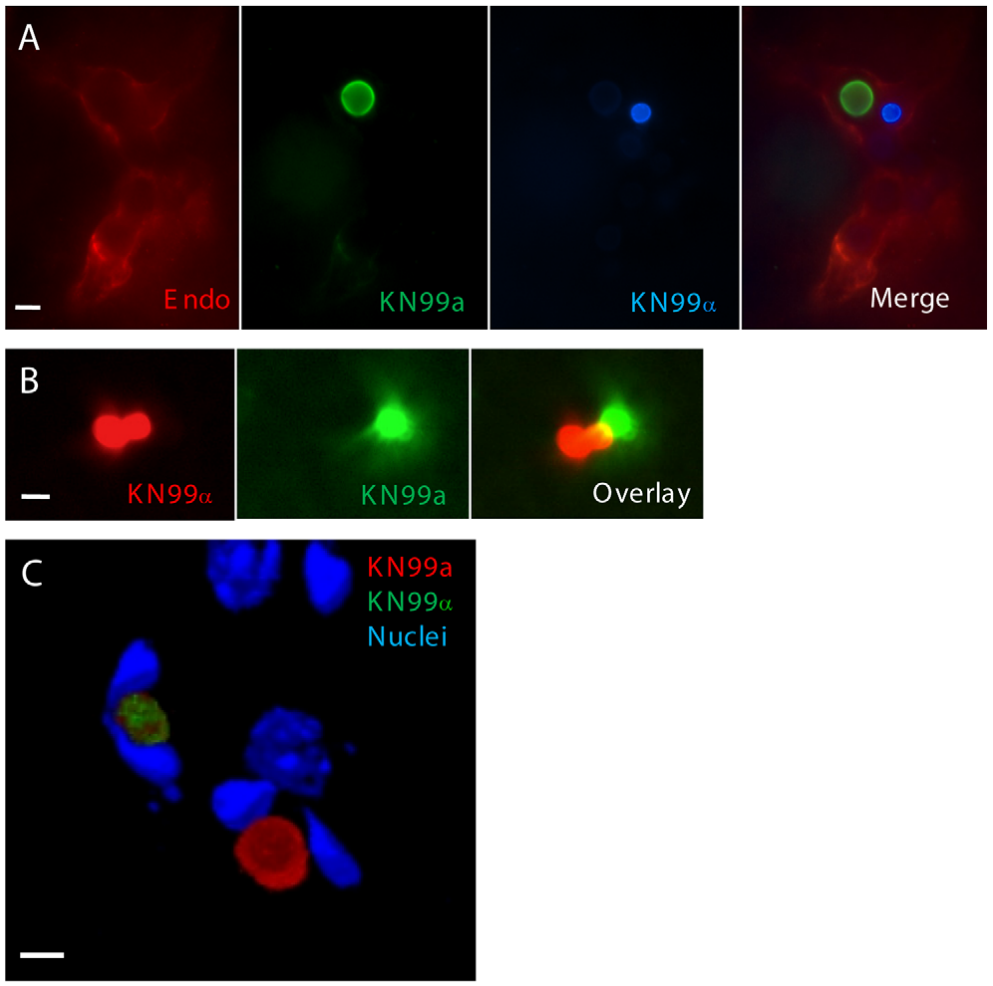
KN99a and KN99α cells interact with endothelial cells of the blood-brain barrier during coinfection. **a** or α were combined with AlexaFluor 350 (blue), AlexaFluor 488 (green) or AlexaFluor 594 (red) and incubated for 20 minutes. Mice were inoculated by tail vein injection with an approximate 1∶1 ratio of **a**:α at a final concentration of 2×10^7^ cells. At 1 day post-infection, animals were sacrificed and 50 µm frozen brain sections were obtained. **A**) Sections from mice infected with **a** (green) and α (blue) were immunostained with anti-collagen IV primary antibody (endothelial cell membrane) with a TRITC (red) labeled secondary antibody. Bar  = 20 µm **B**) Sections from mice infected with **a** (green) and α (red) were imaged by confocal microscopy and sections were compiled as a projection. Bar size  = 20 µm **C**) Frozen sections from mice infected with **a** (red) and α (green) were treated with Hoechst (host cell nuclei), imaged by confocal microscopy, and sections were compiled as a 3D rendering. The U-shaped nuclei are indicative of endothelial cells containing cryptococcal cells. Bar  = 10 µm.

An *in vivo* murine tail vein injection model was employed to determine whether coinfection disrupts CNS penetration by reducing **a** cell interactions with the endothelial cells of the BBB [34], [35]. In this model, cells bypass the lungs and are injected directly into the bloodstream via the mouse tail vein. The cells then lodge in the small capillaries of the brain and cross the endothelial cell layer of the BBB. To test whether interaction with the BBB was directly affected by mating type or coinfection, **a** and α cells were fluorescently labeled and examined for their interactions with the BBB. Both cell types were able to traffic to the small capillaries of the brain (**Figure 4A**) and quantification revealed equal proportions of the two mating types in the capillaries (data not shown). During coinfection, the two mating types were observed in close proximity approximately 25% of the time, consistent with random interactions between cells in a mixed population. The finding that cells of opposite mating type are found in close association would enable pheromone signaling to occur between them in the capillaries of the brain (**Figure 4B**). Both mating types could induce phagocytosis by the endothelial cells of the BBB (**Figure 4C**). Capsule structural changes are important for interactions with the endothelial cells of the BBB [35]. These structural changes can be characterized by alterations in anti-capsular antibody binding. The binding patterns to the cryptococcal capsule for two monoclonal antibodies, E1 and CRND-8, recognizing distinct epitopes on the capsular polysaccharide were studied over time and found to be similar for both mating types. Cells observed in the capillaries shortly after inoculation and up to 6 hours post-infection exhibited only E1 antibody binding. In contrast, cells observed in the brain parenchyma were mostly labeled with CRND-8, as described previously for KN99α [35]. No difference in the capsular antigen staining or the kinetics of capsular changes upon crossing of the BBB were observed between the **a** and α cells during interactions with the endothelial cells of the BBB – either alone or during coinfection (data not shown). These data suggest that the inability of **a** cells to penetrate the CNS during coinfection is not due to innate differences between the two cell types or their interactions with the BBB itself, but instead may be due to an inability of the **a** cells to traffic appropriately from the lungs to the brain.

### Titan cells are resistant to phagocytosis

One of the first lines of defense by the host immune system is phagocytosis and the resultant killing of pathogens by mononuclear macrophages and monocytes in the lungs. These host cells identify pathogens, phagocytose them, and either kill the pathogen outright via oxidative and/or nitrosative bursts or present antigens to T cells for further activation of the host immune response [36]. Recent studies suggest phagocytosis by monocytes or macrophages is important for subsequent CNS penetration [16], [18], [20], [22]. Thus, we examined titan cell interactions with lung host immune cells.

**Figure 5:**
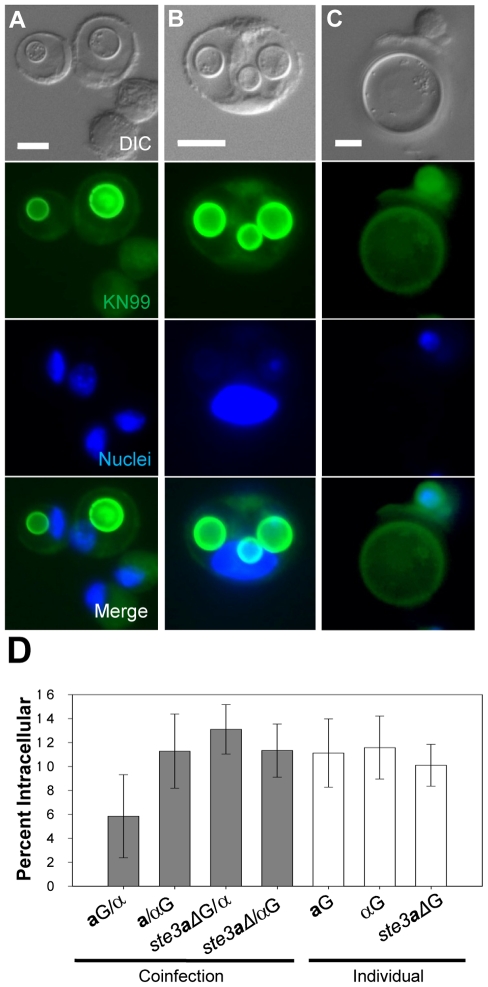
Titan cell formation and phagocytosis in the lungs of infected mice. Mice were intranasally infected with either **a**, α, or *ste3* ***a*** *Δ* cells labeled with AlexaFluor 488 (green) or coinfected with one labeled and one unlabeled strain (four mice per treatment). Cells obtained by BAL were fixed, stained with DAPI, and examined by microscopy for green fluorescence (cell type) and cell size. >500 cells were examined per animal. Bar  = 10 µm **A**) *C. neoformans* **a** cells (green) ≤10 µm in diameter were visible inside host phagocytes. Host cells were identified by large blue DAPI stained nuclei. **B**) Several small α (≤10 µm) cells (green) can be seen inside a single host cell. **C**) Mating type **a** titan cells (>10 µm) are seen in contact with host phagocytes but are too large to be phagocytosed. **D**) Cells obtained by bronchoalveolar lavage (BAL) were fixed and examined by microscopy for green fluorescence and percent phagocytosis. >500 cells were examined per animal. Error bars indicate SD from four mice per treatment. Asterisk indicates p<0.09 in pair-wise comparisons to all other samples; p-values >0.4 were observed for other pair-wise comparisons.

Fixed BAL samples were analyzed microscopically for yeast cell interactions with host phagocytic cells. Titan cells were never observed inside host phagocytes, presumably due to their large size. Engulfed small cryptococci were observed inside phagocytic host cells (**Figure 5A, B**). No difference in phagocytosis was observed between mating types (p = 0.82, **Figure 5D**). The percentage of intracellular α cells during coinfection was similar to that observed in single mating type infections (p>0.89, **Figure 5D**). In contrast, a decrease in the percentage of phagocytosed **a** cells was seen during coinfection (p<0.09, **Figure 5D**). Phagocytosis was restored in the *ste3* ***a*** *Δ#1* mutant (p>0.53 **Figure 5D**). Interestingly, titan cells were often surrounded by one or more host immune cells (**Figure 5C**). Yet complete phagocytosis of titan cells was not observed upon characterization of these cellular interactions by confocal microscopy (data not shown). Taken together, these data indicate titan cell formation was negatively correlated with phagocytosis by host immune cells.

**Figure 6:**
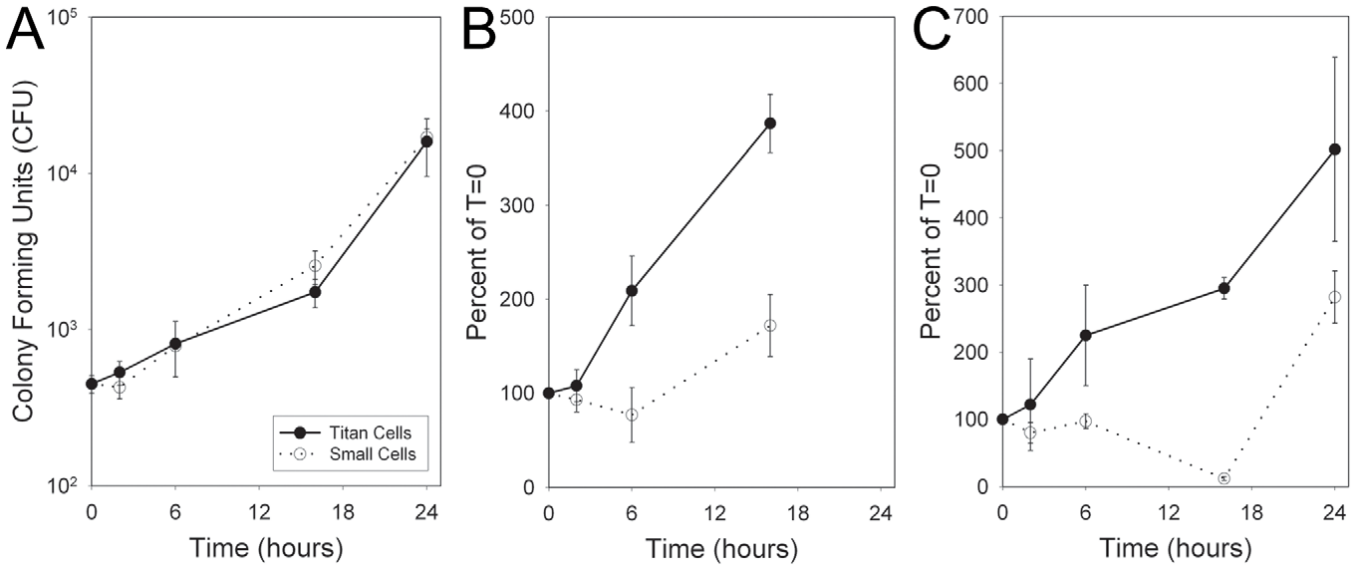
Titan cells are resistant to oxidative and nitrosative stress. Mice were coinfected intranasally with 4×10^7^ cryptococcal cells. At 3 days post-infection, BALs were performed and cells were sorted by FACS based on size. 2×10^4^ titan cells or small cells were resuspended in 100 µL RMPI. Cryptococcal cells received **A**) no treatment, **B**) 10 mM NaNO_3_, **C**) 1 mM TBHP. At 0, 6, 16, or 24 hours, aliquots of each treatment were plated on YPD agar and colony forming units (CFU) were determined. Error bars indicate SD from three replicates.

Both macrophages and neutrophils employ oxidative and nitrosative bursts as a means of killing pathogens (Janeway et al., 2008). Titan cell resistance to these stresses was characterized by comparing the growth of purified titan and small cells isolated by cell sorting of BAL samples. Both cell types showed equivalent growth in the absence of oxidative or nitrosative stress (**Figure 6A**). Treatment with sodium nitrate (NaNO_3_) slowed the growth of the small cell population compared to the titan cell population (**Figure 6B**). Treatment with tert-butyl hydroperoxide (TBHP) resulted in killing of small cells, represented by a decrease in cell counts relative to the initial time point (**Figure 6C**). In contrast, titan cells exhibited continued growth in the presence of these oxidative stresses (**Figure 6C**). Similar results were observed with stabilized hydrogen peroxide treatment. Thus, titan cells are more resistant than normal cells to both oxidative and nitrosative stresses similar to those employed by cells of the host immune system.

### Titan cells are polyploid

In yeasts, cell enlargement is often associated with either cell cycle arrest or increased DNA content [37], [38], [39]. Pheromone sensing in the model yeasts *Schizosaccharomyces pombe* and *Saccharomyces cerevisiae* is known to trigger a cell cycle arrest. We examined titan cells for their progression through the cell cycle by characterizing their ability to bud and produce daughter cells. In addition, we determined the DNA content of titan cells.

**Figure 7:**
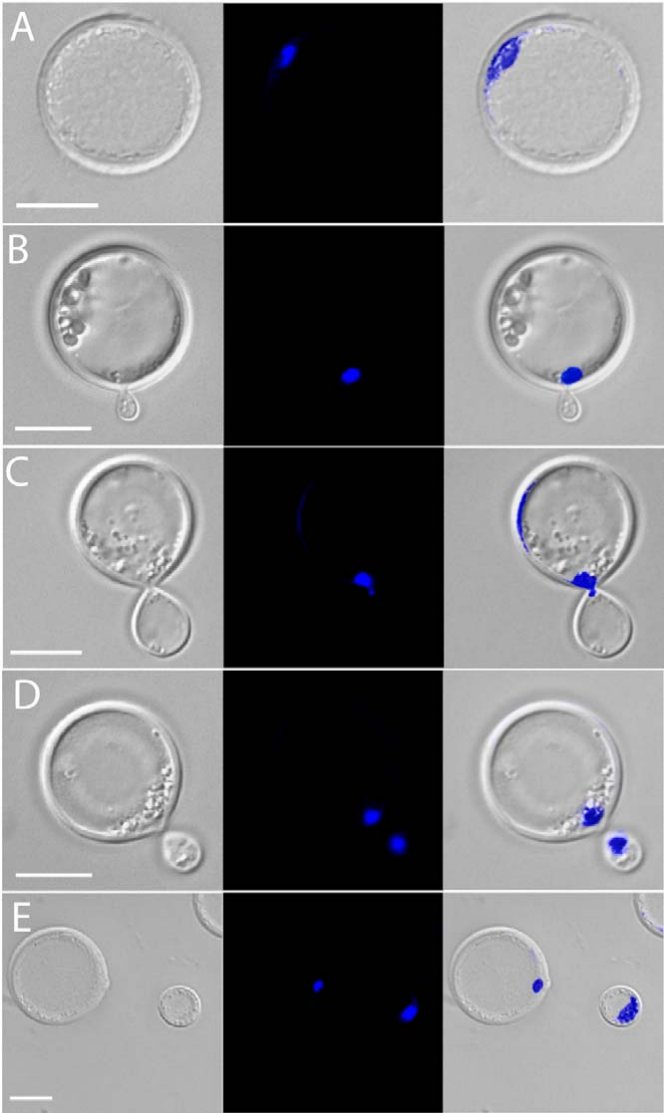
Titan cells can undergo cell division. Mice were infected with 5×10^7^ cells by inhalation of an approximate 1∶1 ratio of **a:**α cells. At 3 days post-infection, mice were sacrificed, BALs performed, and the resulting cells were fixed and DAPI stained for nuclear content. **A**) Titan cell containing a single nucleus. **B**) Titan cell early bud formation. **C**) Nuclear transfer from a mother (titan cell) to a daughter cell. **D**) Titan cell late bud formation. **E**) Cytokinesis of a daughter cell from a titan cell. Bar  = 10 µm.

Titan cells produced *in vivo* were obtained from BAL of mice with single or coinfections. The cells were immediately fixed and stained with DAPI. Microscopic examination of titan cells revealed a single nucleus (**Figure 7A**). Analysis of titan cell nuclear structure by confocal microscopy and z-stack sectioning showed the nucleus had an elongated tubular shape instead of the classic round shape observed in smaller cells (data not shown). Because of its elongated shape, only a portion of the nucleus was observed in each focal plane. Several stages of the cell cycle were identified. In early bud formation (**Figure 7B**), titan cells had a nucleus in the mother cell while the daughter cell lacked a nucleus. The mother cell nucleus was observed at the bud site and entering the daughter cell (**Figure 7C**). After nuclear division the mother and daughter each contained single nuclei (**Figure 7D**). Finally, after cytokinesis was complete, individual nuclei were visible in the mother and associated daughter cell (**Figure 7E**). Budding of the titan cells was readily observed from *in vivo* samples suggesting complete cell cycle arrest would not explain the increased titan cell size.

Increases in cell size in plants, or gigantism, is often correlated with increased ploidy [40]. Because titan cells contain only one nucleus, we quantified their DNA content by flow cytometry and quantitative PCR. Fluorescently labeled cells from individual or coinfections were isolated by BAL and immediately fixed and stained with DAPI. The fixed cell suspensions were then analyzed using an imaging flow cytometer to define cell populations (**Figure S3**). Two distinct populations of fluorescent cryptococcal cells were identified: cryptococcal cells alone and cryptococcal cells inside host cells. Because phagocytosed cryptococcal cell size cannot be accurately measured with flow cytometry, only single non-phagocytosed yeast cells were examined further. The single non-phagocytosed yeast cells were then divided into three populations based on cell diameter: ≤10 µm, >10 µm but ≤20 µm, and >20 µm. The ≤10 µm cell population was designated as small cells of typical size for *Cryptococcus*. The group of cells >20 µm were designated as the titan cell population. The intermediate cell population, >10 µm but ≤20 µm, contained a mixture of small and titan cells, thus could not be accurately characterized by flow cytometry.

**Figure 8:**
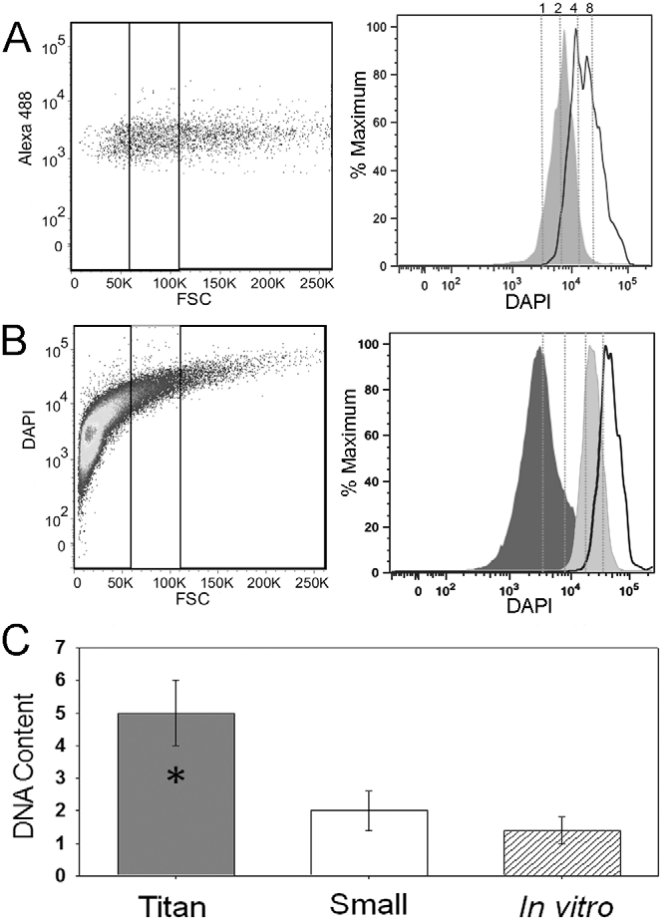
Titan cells have increased DNA content. Mice were intranasally infected with 5×10^7^ cells with an approximate 1∶1 ratio of **a:**α cells labeled with AlexaFluor 488 (green). At 3 days post-infection, mice were sacrificed, BALs performed, and the resulting cells were fixed. **A**) Cells were stained with DAPI to measure nuclear content by flow cytometry. Left panel indicates small (≤10 µm) and titan (>20 µm) cell population gates. Right panel indicates DNA content based on DAPI fluorescence for small (dark gray) and titan (white) populations normalized to cell number (% maximum). Dashed lines indicate predicted 1C, 2C, 4C, and 8C DNA content based on DAPI intensity of the 1C and 2C control cells stained and analyzed in the same experiment. **B**) Cells were grown *in vitro* in spent DMEM liquid medium for 7 days at 30°C. Cells were fixed and stained with DAPI. Left panel indicates small (≤10 µm), intermediate (>10, ≤20 µm), and titan (>20 µm) cell population gates. Right panel indicates DNA content based on DAPI fluorescence for small (dark gray), intermediate (light gray), and titan (white) populations normalized to cell number. Dashed lines indicate predicted 1C, 2C, 4C, and 8C DNA content based on DAPI intensity of the 1C and 2C control cells strained and analyzed in the same experiment. **C**) Fixed BAL samples were sorted into small and titan cell populations by fluorescence activated cell sorting. DNA was purified from the sorted populations, normalized to cell number, and chitin synthase 1 (*CHS1*) gene copy number was determined by comparison to a log phase control sample with a known ratio of 1C:2C cells with a total gene copy number equivalent to 1.4.

Flow cytometry and cell sorting of 50,000 cells were used to obtain an accurate representation of the DNA content for each population (**Figure 8**). DNA content determinations were based on DAPI fluorescence in haploid cells grown *in vitro* in Dulbecco's modified eagle medium (DMEM) at 37°C and 5% CO_2_ (non-titan-inducing conditions) (**Figure S4**). The small cell population isolated from coinfected mice showed a prominent peak consistent with a majority of the cells in the population containing two copies (2C) of DNA. These data would suggest that most of the small cell population *in vivo* were in G2 of the cell cycle (**Figure 8A**). In contrast, the titan cell population showed two peaks consistent with 4C or 8C DNA content (**Figure 8A**). No differences in titan cell DNA content were observed between the two mating types or in individual versus coinfections, indicating that titan cell DNA content was not altered by coinfection (**Figure S4**).

Analysis of the *in vivo* samples suggested that both the small cells and titan cells could be undergoing active cell growth and replication, making characterization of titan cell ploidy difficult in these *in vivo* samples. To determine the ploidy of titan cells, we identified *in vitro* conditions that stimulated titan cell production. Titan cell formation was only observed in cryptococcal samples grown in spent media previously used to culture mammalian cells (**Figure S5**). Differences in titan cell formation were observed based on the media used, the temperature of incubation, and mammalian cell type. Optimal *in vitro* titan cell production was observed when cryptococcal cells were grown in spent DMEM derived from MH-S alveolar macrophages at 30°C. When grown to stationary phase for 5 days in this medium approximately 4% of the total population was titan cells. On average, titan cells generated *in vitro* were smaller than those observed *in vivo*, ranging from 15 µm to 30 µm in diameter. Due to the smaller size of the *in vitro* titan cells, the intermediate cell population (>10 µm but ≤20 µm) was included in the flow cytometric DNA content analysis (**Figure 8B**). In contrast to the *in vivo* samples, the DNA content of the *in vitro* small cell population at 5 days was consistent with 1C cells, suggesting that the cells were in stationary phase (**Figure 8B**). Cells grown to stationary phase in a standard growth medium were also 1C (data not shown). The intermediate cell population had a single peak consistent with 4C cells and the larger titan cell population (>20 µm) had a single peak consistent with 8C cells (**Figure 8B**). Thus, titan cells in stationary phase appeared to be either tetraploid or octoploid based on cell size.

Quantitative PCR was used to determine the average copy number per cell of the chitin synthase 1 (*CHS1*) gene as an additional molecular characterization of DNA content in the *in vivo* small and titan cell populations. Quantitative PCR was performed on the isolated DNA from three cell populations (small, titan, control). This quantitative PCR analysis confirmed that the titan cells had increased *CHS1* DNA content compared with the small cells (p<0.001, **Figure 8C**). Consistent with the flow cytometry results, the small cells had an average *CHS1* gene copy number of 2 in the *in vivo* samples, suggesting that the majority of the population had a 2C DNA content. The titan cell population had an average *CHS1* gene copy number of 5, consistent with a 3∶1 ratio of 4C to 8C cells. Taken together, the *CHS1* gene copy number and flow cytometry data suggest that titan cells are tetraploid and octoploid.

## Discussion

We characterized a novel cell morphology produced by *C. neoformans*, referred to as “titan” cells. These enlarged cells have been observed in the lungs of mice following intranasal instillation [29] and can be up to 100 µm in diameter. Titan cells are commonly seen in human clinical isolates [30], [32]. As early as the 1970s, cells greater than 50 µm in diameter were observed in sputum samples of an infected patient [30]. Our studies demonstrate that titan cells are resistant to oxidative/nitrosative stresses and phagocytosis by host macrophages. We propose that alterations in phagocytosis are beneficial to cell survival in the lungs early in the infectious process but impede dissemination to the major site of disease in the brain.

One fifth of the cells in mouse lungs were titan cells following an initial pulmonary infection. The level of titan cell production varied depending on inoculum size. Inoculation with 5×10^4^ cryptococcal cells resulted in almost 30% titan cell formation. In contrast, inoculation with 5×10^6^ cryptococcal cells resulted in approximately 15% titan cell formation. Differences in titan cell formation in response to cryptococcal cell density/burden in the lungs were also observed by Zaragoza and colleagues [41].

Titan cell formation was stimulated by coinfection with strains of opposite mating type. Analysis of titan cell formation in the two mating types revealed that only **a** cells increased titan cell production upon coinfection. Concomitant with this increased titan cell formation we observed a decrease in **a** cell accumulation in the brain. Interestingly, **a** cell hematogenous dissemination to other organs, such as the spleen, was unaffected by increased titan cell formation. Our molecular studies implicate the pheromone response MAPK signal transduction pathway as a regulator of titan cell production. The increase in titan cell formation and reduction in CNS penetration during coinfection was dependent upon the Ste3**a** pheromone receptor. Mutant strains lacking Ste3**a**, and therefore unable to sense the presence of pheromone, did not enhance titan cell formation during coinfection and exhibited BBB penetration equivalent to α strains. The clinical relevance of *in vivo* pheromone signaling and its effect on the infectious process cannot be determined without first understanding the prevalence of coinfections in humans. Irrespective of the biological significance of pheromone signaling, alteration of titan cell formation using the pheromone signaling pathway is a powerful tool to dissect the effect of titan cell formation on disease progression.

The *ste3* ***a*** mutant strain exhibited only a slight decrease in the basal level of titan cell formation in the absence of α cells, suggesting that pheromone sensing is not the only pathway leading to titan cell production. The observation that pheromone signaling only modifies the level of titan cells suggests that identification of other signaling pathway(s) involved in titan cell formation will be key to understanding other biologically relevant signals that trigger titan cell formation.

The observation that titan cells can be generated *in vitro* by culture in spent medium suggests that cryptococcal cells may sense the presence or absence of a compound in this medium. Titan cell production was predominantly stimulated by spent media from a macrophage cell line. In contrast, little titan cell production was observed in spent media from an endothelial cell line, suggesting the compound could be cell-type specific. An increase in cell size has also been observed in mice deficient in T cells and NK cells [22]. Differences in titan cell formation were also observed in different media and at different temperatures. Thus, we cannot rule out the possibility that the signal is an absence or unavailability of specific nutrients. Taken together, these data suggest there could be four or more signals leading to titan cell formation: host, temperature, nutrients, and pheromone.

**Figure 9:**
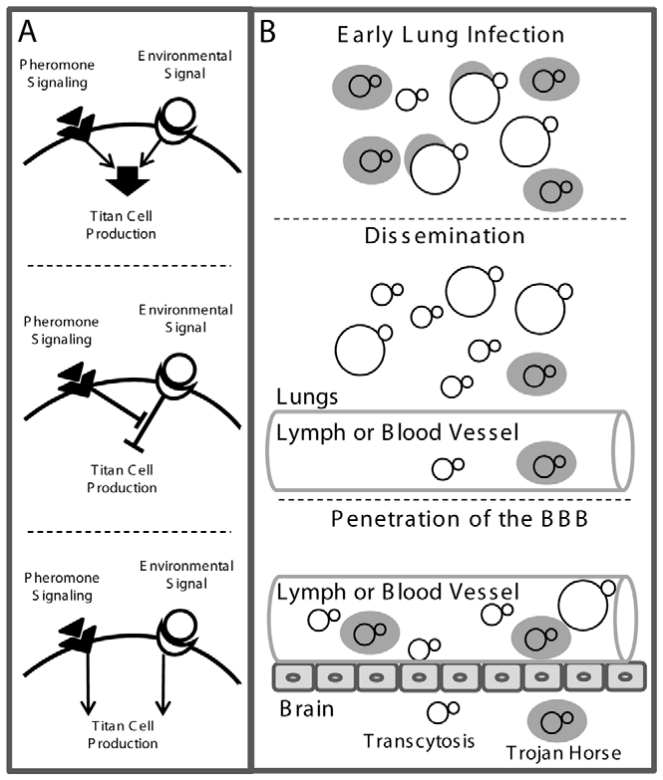
Model of titan cell signal transduction and pathogenesis. **A**) Putative pathways involved in titan cell production. *Top:* Pheromone signaling may enhance an environmental sensing signal leading to titan cell production. *Middle:* Pheromone signaling may inhibit a negative regulator of titan cell formation. *Bottom:* Pheromone signaling may act independently of the environmental sensing and/or other pathways leading to titan cells formation. **B**) The effect of titan cell formation on pathogenesis. *Top:* Upon initial infection, titan cell production protects a subset of cells from phagocytosis and killing, to establish the infection. *Middle:* *C. neoformans* cells disseminate from the lungs either as free cells or inside host phagocytes. *Bottom:* Phagocytosis is required for efficient penetration of the blood-brain barrier. Cryptococcal cells may enter the brain by transcytosis, or as a “Trojan horse” inside of host phagocytes. Over-production of titan cells reduces blood-brain barrier penetration as a result of decreased phagocytosis.

At least three possibilities could account for pathway interactions affecting titan cell formation (**Figure 9A**). First, the pheromone signaling pathway may positively affect an environmental sensing pathway to increase the signal leading to titan cell production (**Figure 9A** **top**). Second, pheromone signaling may inhibit a negative regulator of titan cell formation (**Figure 9A** **middle**). Finally, the pheromone signaling pathway may be independent of the environmental sensing pathway leading to titan cell formation (**Figure 9A** **bottom**). Mutant analysis has revealed that signaling pathways such as PKA, cAMP, and RAS can affect cell size *in vitro* [31], [41], [42] and may be involved in the environmental sensing or pheromone signaling pathways leading to titan cell formation.

Titan cells have higher DNA content compared with smaller cells. Titan cell production may be a result of a cell cycle pause or increased DNA replication due to other mechanisms. In *S. cerevisiae* the cell cycle mutant, *cdc24*, can be induced to produce yeast cells up to six times greater in volume than normal cells due to continued growth in the absence of cell division [39]. However, because titan cells are able to continue producing daughter cells, it is unlikely they are generated by a complete cell cycle arrest, as in the *cdc24* mutant. An increase in DNA content from haploid to tetraploid has been implicated in increased cell size and morphology changes in *S. cerevisiae* [43]. This may be due to an increase in transcripts that regulate passage from G1 to S phase in the tetraploids [43]. *C. neoformans* titan cells do not appear to have the mitosis defects and lowered viability seen in *S. cerevisiae* tetraploid cells [44], [45]. These data suggest *C. neoformans* may have a distinct cell cycle regulation that allows titan cell replication. An increase in DNA content may be necessary to generate and sustain titan cells. Zaragoza and colleagues [41] have shown that titan cells have altered capsule formation and cell wall composition. These processes, along with the need to sustain a large cell, may require increased transcriptional and translational capacity by the cell. Additional copies of DNA may facilitate this process.

An alternative hypothesis is that the increase in cell size protects against the host immune system and that the increase in DNA content promotes rapid generation of daughter cells. This phenomenon is observed in other human pathogenic fungi. *Pneumocystis* and *Coccidioides* species also exhibit increases in cell size and nuclear content in the lungs of infected hosts. In *Pneumocystis*, many cells are in a trophic haploid state and reproduce by binary fission, yet a subset of cells is thought to undergo a sexual cycle to produce enlarged cysts [46], [47]. It is hypothesized that the *Pneumocystis* sexual cycle involves fusion of two haploid trophic cells to produce a diploid cell. Although the exact signal for fusion is unknown, molecular analysis has revealed a MAPK pathway similar to the *C. neoformans* pheromone signaling pathway, including a homolog of the Ste3 pheromone receptor [48], [49]. The resulting diploid cell undergoes meiosis followed by mitosis, forming a cyst containing eight nuclei [46], [47]. As the *Pneumocystis* cyst matures, the nuclei develop into new trophic cells and are released into the surrounding environment [46], [47], [50]. In contrast to cryptococcal titan cells, *Pneumocystis* cyst formation produces β-glucan resulting in increased recognition and phagocytosis by host immune cells [47].

*Coccidioides immitis* also undergoes dramatic morphological changes *in vivo* [4], [51]. Infection begins with inhalation of spores, or arthroconidia, from the environment that are roughly 2 to 4 µm in diameter. The arthroconidia produce round cells in which nuclear division gives rise to multinucleated cells called spherules [4], [51], [52], [53]. The developing spherules range in size from 60 to100 µm or larger. Host immune cells, such as macrophages and neutrophils, are unable to phagocytose the *Coccidioides* spherules due to their large size, protecting the cell from destruction while it generates endospores [4]. Eventually the spherule ruptures, releasing the endospores, and the cycle starts again. In *C. neoformans*, titan cells are also protected from phagocytosis. Titan cells do not rupture but instead produce daughter cells both *in vitro* and *in vivo* by budding. Unlike *Pneumocystis* and *Coccidioides* where enlarged cells contain multiple nuclei, the cryptococcal titan cells appear to have a single nucleus. Interestingly, titan daughter cells are small, suggesting they may be haploid. These findings imply ploidy changes occur both during formation of titan cells and their daughter cell progeny. Ploidy changes can occur via sexual, parasexual, and endoreplicative processes. Further studies are necessary to determine the method by which ploidy changes occur in *C. neoformans*.

Titan cell production was observed within 1 day post-infection, yet the proportion of titan cells present in the lungs plateau by 7 days post-infection and remain constant throughout the rest of the infection. The observation that titan cell production is only stimulated at early stages of infection implies titan cells may promote pathogenesis early in the infectious process but are dispensable later. *Cryptococcus* is acquired from the environment by inhalation of spores or desiccated yeast cells. Previous studies examining changes in transcription and subsequent up-regulation of virulence factors in response to changes in temperature or phagocytosis by macrophages show that the initial population of inhaled cells is unlikely to be prepared for survival and replication in the host [8], [54], [55], [56], [57]. Thus, the vast majority of cells in the initial inoculum are likely to be engulfed and destroyed by host mononuclear phagocytes. Titan cell production protects a subset of cells from phagocytosis, possibly due to increased size. While not tested directly in this study, the observed *in vitro* survival in the presence of oxidative/nitrosative stresses may also promote titan cell resistance to killing mechanisms utilized host immune cells. Thus, the titan cells are able to survive the initial host immune response.

Pheromone-mediated titan cell production did not increase the prevalence of **a** cells in the lungs over time. These data show that titan cell formation does not enhance persistence in the lungs. In support of this conclusion, titan cell formation is not readily observed in the rat persistence model of cryptococcosis [58], [59], [60], [61], [62], [63], [64]. Yet, the *C. neoformans* var. *neoformans* strain most commonly used in the rat model readily generates titan cells in mice [41], [63]. Because titan cells are readily observed in human tissue, where dormancy is thought to be important in the infectious process [11], [65], [66], we cannot rule out the possibility that titan cells play a role in dormancy and/or reactivation in human infections.

Dysregulation of titan cell production in the coinfection model did not affect hematogenous dissemination from the lungs to the spleen; yet increased titan cell formation was correlated with a significant decrease in dissemination to the CNS. Survival in macrophages has been shown to be important for trafficking to the CNS [18], [19]. Additionally, recent studies have demonstrated that phagocytosed cryptococcal cells are more efficient at disseminating to the CNS than non-phagocytosed cells [16], [20]. Thus, interactions with host phagocytes promote cryptococcal BBB penetration and subsequent neurological disease. Increased titan cell formation in the lungs during coinfection reduced phagocytosis which subsequently inhibited dissemination to the CNS. It is still unclear whether coinfections are a common occurrence in *Cryptococcus* pathogenesis, yet the effect of coinfection on titan cell production has allowed us to study this important morphological transition. Our data support a model in which titan cells are advantageous at early stages of the infection (**Figure 9B**). The ability of titan cells to evade phagocytosis allows *C. neoformans* to establish the initial lung infection and overcome the initial immune response generated by resident macrophages (**Figure 9B top**). Because severe cryptococcal infections are often seen in patients with T cell deficiencies, it is likely that the clearance of the initial infection is T cell mediated. (**Figure 9B middle**). Our model predicts that phagocytosis of the daughter cells allows dissemination to the CNS, resulting in neurological disease (**Figure 9B bottom**).

Examples of cell morphology changes and cell surface alterations that are important in the infectious process can be found throughout the microbial world. In the protozoan pathogen *Toxoplasma gondii*, cysts are formed in response to elements of the host immune response, including IFNγ, pH changes, and nitrosative stress. These cysts are able to escape immune recognition and establish an asymptomatic chronic infection [67], [68]. Uropathogenic *Escherichia coli* strains are known to undergo filamentation in response to TLR4 signaling by host immune cells [69]. During development of these bacterial colonies, a subset of the cells filament. The filaments are then able to evade phagocytosis by host neutrophils that are recruited to the area in response to infection [69]. Reovirus undergoes dramatic morphological changes in the host. The reovirus virion can be degraded by proteases revealing a stable intermediate subvirion particle (ISVP). Although both viral morphologies are infectious, the virion is more restricted in host range, while the ISVP can infect a wider variety of cell types [70], [71], [72].

Similar to morphological changes in other microbes, titan cell formation alters the host-pathogen interaction in the lungs during early cryptococcal infection. By studying these host-pathogen interactions and the molecular triggers involved in *C. neoformans* titan cell production, we may gain general insight into how morphological changes can affect pathogenicity of microbes. Our studies highlight the complex morphological variations microbes deploy to avoid recognition and killing by the host immune system.

## Materials and Methods

### Ethics statement

All animals were handled in strict accordance with good animal practice as defined by the relevant national and/or local animal welfare bodies, and all animal work was approved by the appropriate committee. Experiments at the University of Minnesota were reviewed and approved by the university Institutional Animal Care and Use Committee (IACUC) under protocol number 0712A22250. Experiments at the University of North Carolina – Chapel Hill were reviewed and approved by the university IACUC under protocol number 09-166.0. Studies at the Institut Pasteur we reviewed and approved under protocol number CHSCT#03-344.

### Strains and media

The congenic *C. neoformans* var. *grubii* strains KN99**a** and KN99α were used in this study [27]. Strains were stored as glycerol stocks at −80°C and grown at 30°C in yeast extract-peptone-dextrose (YPD) agar or broth medium (BD, Hercules, CA).

### Tissue analysis

*C. neoformans* cells were cultured overnight in YPD broth. The resulting yeast cells were pelleted and resuspended in sterile phosphate-buffered saline (PBS) at a concentration of 1×10^6^ cells/ml based on hemocytometer count. Groups of 6- to 8-week-old female A/J mice (Charles Rivers, NCI, Frederick, MD; Jackson Labs, Bar Harbor, MA) were anesthetized by intraperitoneal pentobarbital injection. Three mice per treatment per time point were infected intranasally with 5×10^4^ KN99**a**, KN99α, or an approximate 1∶1 mixture of KN99**a**:KN99α cells in 50 µl PBS. The concentration of cells in the inoculum was confirmed by plating serial dilutions and enumerating colony forming units (CFU). At 1, 2, 3, 7, 14, or 21 days post-infection mice were sacrificed by CO_2_ inhalation. The heart, lungs, brain, kidneys, liver, and spleen were harvested, fixed in 10% buffered formalin, paraffin-embedded, sectioned, and stained with PAS (periodic acid Schiff) or H&E (hematoxylin and eosin). Tissue sections were examined for cell size and morphology by microscopy. To examine fluorescently labeled cells in tissue sections, the yeast cells were incubated with AlexaFluor 350 (blue), AlexaFluor 488 (green), or AlexaFluor 594 (red) succinyl esters for 10–20 minutes at 25°C using the appropriate Protein Labelling Kit (Invitrogen, Carlsbad, CA). Labeled cells were washed >3 times in sterile PBS to remove unbound dye. The cells were resuspended in PBS at a concentration of 1×10^8^ based on hemocytometer count. Three mice per treatment (**a**, α, or coinfection) were infected intranasally with 5×10^6^ fungal cells. The concentration of yeast cells in the inoculum was confirmed by plating serial dilutions and enumerating CFU and the proportion of **a** cells in the coinfection inoculum was determined by mating assay [27]. Infected mice were sacrificed at 1, 2, or 3 days post-infection by CO_2_ inhalation. Lungs were extracted and fixed as described above and unstained sections were examined for cell size, morphology, and fluorescence. Data presented are representative of three independent experiments with two or three mice per treatment per experiment.

### *ste3aΔ* mutant strains

Two independent ste3**a**Δ mutant strains were generated by gene disruption as previously described [73].The nourseothricin transgene (NAT) was used to replace the *STE3* ***a*** gene coding region. PCR was used to generate the 5′ (KN0035 and KN0036) and 3′ (KN0037 and KN0040) flanking regions containing linkers to a NAT^r^ cassette and overlap PCR generated the NAT insertion allele (**Table 1**). The mutant allele was introduced by biolistic transformation into KN99**a** to generate *ste3* ***a*** *Δ#1* and into the spontaneous ura- strain JF99**a** [74] to generate *ste3* ***a*** *Δ#2*. Transformed colonies resistant to nourseothricin (100 µg/ml) were identified by PCR amplification and sequencing of PCR products spanning a region upstream of the 5′ flanking region into the NAT cassette (KN0079 and KN0031) and from the NAT cassette to downstream of the 3′ flanking region (KN0032 and KN0109). Gene deletion was further confirmed by mating the mutant strains with KN99α on V8, pH 5 media for >14 days at 25°C in the dark. The mutant strains were sterile. The *ste3* ***a*** *Δ#2* was passaged on SD-ura media to isolate a URA+ revertant for use in virulence tests.

**Table 1:** PCR Primers.

Primer Designation	Sequence
**STE3a Knockout construct**	
KN0035	GCCCTAGCAATGTCGATACCC
KN0036	AGCTCACATCCTCGCAGC GCACGTCCGGAGTACACG
KN0032	GCTGCGAGGATGTGAGCT
KN0031	GGTTTATCTGTATTAACACGG
KN0037	CCGTGTTAATACAGATAAACCCTGTATGGCGCTCCTTGGAAG
KN0040	CACAGCAAAGGCACATTCGCAAG
**Outside PCR Checks**	
KN0079	GGAGTTGACGCACGTTTATGGCAA
KN0109	CACTGGTGGAGCATTCATGTCG
**qPCR with primers for CHS1**	
KN104	GTCCCAGGAGGACTCCTTTC
KN105	TGTCGTTCAGGTCGAGTGAG

### *In vivo* analysis of *ste3aΔ* strains

Groups of 5–10 mice were infected with 5×10^4^ cells in an approximate 1∶1 ratio of *ste3* ***a*** *Δ#1*:KN99α, *ste3* ***a*** *Δ#2*:KN99α, or KN99a:KN99αNAT. The actual proportion of **a** cells in the infecting inoculum was determined by growth on selective media. At 21 days post-infection, animals were sacrificed. The lungs, spleen, and brain from each animal were homogenized in 2–4 ml PBS and serial dilutions were plated on YPD for CFU enumeration. >500 colonies per organ were isolated and assayed for antibiotic resistance on YPD containing 100 µg/ml nourseothricin to determine mating type.

### Interactions with the blood-brain barrier (BBB)

KN99**a** and KN99α cells were fluorescently labeled as described above. Three mice per treatment were inoculated by tail vein injection with KN99**a**, KN99α, or an approximate 1∶1 ratio of KN99**a**:KN99α at a final concentration of 2×10^7^ cells. At 1 day post-infection animals were sacrificed, perfused with 20 ml PBS then 20 ml 4% paraformaldehyde (PFA). Brains were harvested, placed in 4% PFA then 40% w/v sucrose solution in PBS, frozen in isopentane and liquid nitrogen, stored at −80°C, and 50 µm sections were generated. For immunohybridizations, slides were washed in PBS for 15 min followed by incubation with 100 µl trypsin-EDTA (Invitrogen) at 37°C for 10 minutes. Slides were then washed in PBS containing 20% fetal calf serum (Invitrogen) for 10 minutes, blocked with PBS containing 20% FCS, 0.1% bovine serum albumin (BSA) and 0.1% triton X-100 (Sigma, St. Louis, MO) for 20 minutes, then washed with PBS containing 0.1% triton X-100. Anti-collagen IV antibody (Santa Cruz Biotechnology, Santa Cruz, CA) was diluted to a 1/50 concentration in PBS with 0.1% BSA and 0.1% Triton X-100. Antibody-treated slides were incubated overnight at 4°C followed by washing in PBS. Cy3 labeled goat anti-rabbit antibody was diluted to a 1/200 concentration and added to the slides. After 5 hours of incubation at 37°C, slides were washed three times in PBS for 15 minutes. Hoechst medium was diluted to a 1/500 concentration and added to the slides for 30 seconds. Slides were washed for 5 minutes in PBS and mounted in Vectashield mounting medium. Capsule antigen staining was as described in Charlier et al., 2005 using the CRND-8 and E1 antibodies. Slides were imaged by fluorescence microscopy (Zeiss Axioplan) or by 2-photon confocal microscopy (Zeiss LSM 510 equipped with a Coherent Mira 900 tunable laser) with sections compiled as a projection or as a 3D rendering.

### Bronchoalveolar lavage (BAL)

Four mice per treatment were infected as described above with 5×10^6^ AlexaFluor 488 labeled KN99**a**, KN99α, and *ste3* ***a*** *Δ*#1, or an approximate 1∶1 ratio of one stained and one unstained strain. Infected mice were sacrificed at 3 days post-infection by CO_2_ inhalation. Lungs were lavaged with 1.5 mL sterile PBS three times using a 20 gauge needle placed in the trachea. For flow cytometry, cells in the lavage fluid were pelleted at 16,000 *g*, resuspended in 3.7% formaldehyde, and incubated at room temperature for 30 minutes. Cells were then washed once with PBS, resuspended in PBS containing 300 ng/ml 4′,6-diamidino-2-phenylindole (DAPI) (Invitrogen), incubated at room temperature for 10 minutes, washed with PBS, and resuspended in PBS. >500 cells per animal were analyzed for size and fluorescence by microscopy (AxioImager, Carl Zeiss, Inc). Confocal microscopy (LSM710, Carl Zeiss, Inc) and z-stack imaging (AxioImager with Apotome, Carl Zeiss, Inc) were used to examine interactions with host mononuclear cells. Images were analyzed using Axiovision and Zen software (Carl Zeiss, Inc). Crescent shaped and other fluorescently-labeled cryptococcal cell fragments (i.e. not round cells) were observed within host mononuclear cells. These cell fragments were not included in the analysis.

### Nitrosative and oxidative stress assays

Twelve mice were intranasally infected with 2×10^7^ cells in 50 µL PBS of an approximately 1∶1 ratio of KN99**a** and KN99α cells. At 3 days post-infection, mice were sacrificed by CO_2_ inhalation and BALs were performed. Cells were sorted by FACS using an iCyt Reflection cell sorter (iCyt, Champaign, IL). Cells were sorted based on size using forward scatter (FSC) into small cell and titan cell populations. Purity of samples was checked by flow cytometry and microscopy. Samples were resuspended in Roswell Park Memorial Institute (RPMI) medium 1640 (Invitrogen) supplemented with 10% fetal bovine serum (FBS) (ATCC, Manassas, VA), 4.5 g glucose/L (BD), 1 mM sodium pyruvate (Invitrogen), 0.01 M HEPES (MP Biomedicals, Solon, OH), 5% penicillin/streptomycin (Invitrogen) and 0.05 mM β-mercaptoethanol (Chemicon) to a concentration of 2×10^4^ cells per 100 µL. Samples were then treated with 10 mM NaNO_3_ (Sigma-Aldrich, St Louis, MO), 3 mM H_2_O_2_ (Walgreens Co., Deerfield, IL), or 1 mM tert-butyl hydroperoxide (TBHP) (Sigma-Aldrich). At 0, 6, 16 or 24 hours post treatment, 10 µL aliquots of each sample were plated onto YPD agar for CFU enumeration.

### Flow cytometry

Fixed BAL samples from 4 mice per treatment were generated as described above and analyzed using an ImageStream imaging flow cytometer and INSPIRE software (Amnis Corporation, Seattle Washington). Briefly, images for 5000 cells per sample were collected and analyzed for single cells (R1), doublets (R0), or aggregates of cells (**Figure S3A**). Only single cells (R1) were used in our analyses because cell aggregates would misrepresent cell sizes. Single cells were further analyzed for AlexaFluor 488 fluorescence and DAPI staining (**Figure S3B**). Due to the high nuclear content of mammalian cells, these cells had extremely high DAPI staining (R2 and R3). Non-phagocytosed yeast cells (R5) we identified based on their low DAPI staining. Visual confirmation of cell size in the flow cytometry images was used to identify small and titan cell populations (R6 and R7), that each gate contained only the target cells, and that no contamination between the populations was observed (**Figure S3C**). Data analysis and gating was performed using IDEAS software (Amnis Corporation). Cryptococcal cells grown *in vitro* in YPD or DMEM to log or stationary phase were used as controls to identify haploid cells (1C) and actively dividing cells (1C + 2C).

To examine titan cell ploidy, fixed BAL samples from 4 mice per treatment were generated as described above. *In vitro* control samples were grown in YPD or Dulbecco's modified eagle medium (DMEM, 37°C, supplemented with 10% fetal bovine serum (FBS) (ATCC), 4.5 g glucose/L (BD), 1 M sodium pyruvate (Invitrogen), 0.01 M HEPES (MP Biomedicals, Solon, OH), 5% penicillin/streptomycin (Invitrogen) and 0.05 mM β-mercaptoethanol (Chemicon) for 6 hours (log phase) or 5 days (stationary phase). Spent DMEM or RPMI was collected from MH-S macrophages after 3–5 days culture at 37°C and 5% CO_2_. Spent endothelial cell (EC) media (complete EGM medium, Clonetics, San Diego, CA, USA) was collected from human umbilical vein endothelial cells (HUVEC) after 3–5 day culture at 37°C and 5% CO_2_. *In vitro* titan cells were grown in filter sterilized spent media at 30°C or 37°C for 7 days. *In vitro* and *in vivo* samples were fixed in 3.7% formaldehyde and stained with 300 ng/ml DAPI in PBS. Autofluorescence of non-DAPI stained fixed titan cells was measured and used to set the baseline for ploidy measurements. Cells were examined for cell size by forward scatter (FCS) and nuclear content by DAPI using an LSRII flow cytometer with FACSDiva software (BD) using gating defined by imaging flow cytometry. FCS cell sizes in each gate were verified by microscopy (Zeiss Axioplan). Data presented are representative of three independent experiments with four mice per treatment. 50,000 cells per treatment were analyzed to determine titan cell formation *in vitro*. *In vitro* titan cell formation was variable from experiment to experiment but trends between treatments remained constant. Data presented are representative of five independent experiments. Because the absolute number of cells in each population and in each mouse differed, the DAPI fluorescence for each population was normalized to the number of cells in that population in order to clearly visualize peaks on a histogram representation of the data (**Figure 8**, **Figure S4**). Cells were examined for cell size by forward scatter (FCS) and nuclear content by DAPI using an LSRII flow cytometer with FACSDiva software (BD) using the gating defined by imaging flow cytometry. FCS cell sizes in each gate were verified by microscopy to identify the ≤10 µm, >10 µm but ≤20 µm, and >20 µm cell populations (Zeiss AxioImager). Data presented are representative of three independent experiments with four mice per treatment. 50,000 cells per treatment were analyzed to determine titan cell formation *in vitro*. *In vitro* titan cell formation was variable from experiment to experiment but trends between treatments remained constant. Data presented are representative of five independent experiments.

### Cell sorting and qPCR

Ten to fourteen mice were infected with 5×10^6^ AlexaFluor 488-stained cells at an approximate 1∶1 ratio of KN99**a**:KN99α, as described above. Infected mice were sacrificed at 3 days post-infection and BALs were performed. BALs were pelleted and resuspended in 0.05% SDS in sterile water for 1 minute to promote host cell lysis. Cells were then fixed in 1 ml PBS containing 1% formaldehyde and incubated for 30 minutes at room temperature with mixing. Samples were incubated in 125 mM glycine for 5 minutes, centrifuged at 1500 *g* for 10 minutes, and the pellets were resuspended in ice cold TBS (20 mM Tris, pH 7.6, 150 mM NaCl) containing 125 mM glycine. Cells were washed once in TBS, resuspended in 1 ml PBS and the cell concentration was determined by hemocytometer count. Cell numbers were adjusted to 10^6^ cells/ml, and 1% BSA was added to the fixed cell suspension. Cells were sorted using a FACSAria fluorescence activated cell sorter (FACS) using FACSDiva software (BD). Small and titan cell populations were isolated by FACS using gating as described above. DNA was isolated from 10^6^ cells from small, titan, and 37°C DMEM (control) cell populations. A portion of the control cell population was DAPI stained and the number of haploid and diploid cells in the population was determined by flow cytometry (**Figure S3**). Small cells were classified as ≤10 µm and titan cells were >10 µm. After sorting, the two cell populations were pelleted and resuspended in lysis buffer (50 mM HEPES, 140 mM NaCl, 1% Triton X-100, 0.1% Sodium deoxycholate, 1 mM EDTA). The cell suspensions were transferred to tubes containing 0.3 mm glass beads and vortexed for six 5 minute cycles at 4°C. The bottoms of the tubes were then pierced with a hot 21-gauge needle. The tubes were placed into 15 ml conical tubes and centrifuged at 1500 *g* for 5 minutes at 4°C. The pellets and supernatants were combined and transferred to new tubes. These mixtures were centrifuged for 10 minutes at 10,000 *g* at 4°C and the supernatants transferred to clean tubes. After a further 5 minute centrifugation, the DNA crosslinks were reversed by adding 200 µl TE (10 mM Tris, pH 7.5, 1 mM EDTA) containing 1% SDS to the clarified supernatants and incubating for 6 hours at 65°C. Samples were then incubated 2 hours at 37°C with 250 µl TE containing 0.4 mg/ml proteinase K. After adding 55 µl 4 M LiCl, the DNA was extracted with 0.5 ml phenol and the DNA was precipitated with 100% ethanol. The DNA pellets were washed with 70% ethanol, dried, and resuspended in TE containing 1.5 µl RNase (Ambion AM22886). Samples were stored at −20°C until analyzed by qPCR with primers KN104 and KN105 for chitin synthase (*CHS1*) (**Table 1**). Gene copy number in the control sample was calculated based on the known number of 1C and 2C cells present in that sample (1.4C) based on flow cytometry. The small and titan cell gene copy numbers were normalized to the control sample.

### Statistical analysis

All analyses were performed using Analyse-It (Analyse-it Ltd., Leeds, England). Wilcoxon rank sum analysis was used to analyze differences in coinfection data and *P*-values <0.001 were considered significant. The Mann-Whitney U test was performed to analyze differences between survival curves and *P*-values <0.001 were considered significant. One-way ANOVA was used to analyze differences in titan cell production or phagocytosis and *P*-values <0.05 were considered significant for titan cell production experiments. *P*-values <0.1 were considered significant for phagocytosis experiments.

## Supporting Information

Figure S1 Titan cell formation in the lungs, spleen, and brain. Mice were intranasally infected with 5×10^4^ **a** or α cryptococcal cells. Lungs, brain, and spleen were collected at 1, 3, 7, 14 or 21 dpi. Samples were fixed in 10% formalin and stained with hematoxylin and eosin (H&E). The percentage of titan cells (>10 µm in diameter) was determined by microscopic examination of >500 cells per sample per mouse. Sufficient cell numbers were unavailable in tissue sections from 1 dpi lungs and 1, 3, 7, and 14 dpi spleen and brain for quantification. Error bars indicate SD from six mice per time point.
(2.29 MB TIF)

Figure S2 *ste3a*Δ survival assays. Mice were inoculated with 5×10^4^ cells of either wild-type **a**, ste3**a**Δ#1 (left) or ste3**a**Δ#2 (right) cells and progression to morbidity was monitored.
(2.43 MB TIF)

Figure S3 Imaging Flow Cytometry. *C. neoformans* **a** and α strains were combined with AlexaFluor 488 (green) and incubated at 25°C for 20 minutes. Cells were washed with sterile PBS to remove excess dye. Mice were inoculated with an approximate 1∶1 ratio of **a**:α cells. At 3 days post-infection animals were sacrificed and BALs were performed. The resulting cells were fixed, DAPI stained, and analyzed using an ImageStream flow cytometer using IDEAS software (Amnis Corporation). **A)** Cells were first examined for single cells (R1). Aggregates and doublets were excluded from further analysis. **B)** The R1 population was analyzed for DAPI intensity (X-axis) and AlexaFluor 488 intensity (Y-axis). DAPIhi host cells and phagocytosed cryptococcal cells (R2 and R3) as well as unstained yeast cells (R4) were excluded from further analysis. **C)** Diameter was used to divide the remaining population, R5, into cells <10 µm (R6) and >10 µm (R7). Samples from four mice per treatment were analyzed and gates determined by consensus among the samples.
(8.29 MB TIF)

Figure S4 *C. neoformans* titan cells are polyploid. **a** and α strains were combined with AlexaFluor 488 (green) and incubated at 25°C for 20 minutes. Cells were washed with sterile PBS to remove excess dye. Mice were inoculated with an approximate 1∶1 ratio of **a**:α cells. At 3 days post-infection animals were sacrificed and BALs were performed. The resulting cells were fixed, DAPI stained and analyzed using an LSRII flow cytometer using FACSDiva software (BD). Fluorescently labeled yeast cells were first identified as 488hi and DAPIlow (left). Forward scatter (FSC) was used to identify small (≤10 µm) and titan (>20 µm) cells. Small (blue line) and titan (red line) cell populations were analyzed for DNA content (DAPI) and normalized for cell number (right). **A–D** coinfections **E–G** individual infections **H)** *C. neoformans* cells were grown for 5 days at 37°C and 5% CO_2_ in DMEM, fixed and DAPI stained. Both 1C and 2C peaks can be seen in this cell population. Absolute levels of DAPI intensity in these control cells varied from experiment to experiment thus were included as internal controls for every experiment.
(2.31 MB TIF)

Figure S5 *In vitro* titan cell production. Cryptococcal cells were grown in spent DMEM (MH-S alveolar macrophages), RPMI (MH-S alveolar macrophages), or endothelial cell media (human umbilical vein endothelial cells, HUVEC) at 30°C or 37°C. Samples were fixed in 3.7% formaldehyde and 50,000 cells per sample were analyzed for cell size (forward scatter). Data presented are representative of five independent experiments.
(2.12 MB TIF)
