# Correction: Cryptococcal Cell Morphology Affects Host Cell Interactions and Pathogenicity

**DOI:** 10.1371/annotation/1b59fd9e-9ac9-4ea8-a083-14c413c80b03

**Published:** 2010-06-23

**Authors:** Laura H. Okagaki, Anna K. Strain, Judith N. Nielsen, Caroline Charlier, Nicholas J. Baltes, Fabrice Chrétien, Joseph Heitman, Françoise Dromer, Kirsten Nielsen

The publisher wishes to note that the "Accept" date on this article reflects the "Production Accept" date. This article was accepted by the editor on 27 January 2010, while its companion article (Zaragoza O, García-Rodas R, Nosanchuk JD, Cuenca-Estrella M, Rodríguez-Tudela JL, et al. (2010) Fungal Cell Gigantism during Mammalian Infection. PLoS Pathog 6(6): e1000945. doi:10.1371/journal.ppat.1000945) was accepted by the editor on 16 April 2010.

