# Supplementary figures and images for: Cryptococcal Cell Morphology Affects Host Cell Interactions and Pathogenicity
Source: PLoS Pathog. 2010 Jun 17;6(6):e1000953. doi: 10.1371/journal.ppat.1000953 (PMC2887476; doi:10.1371/journal.ppat.1000953)

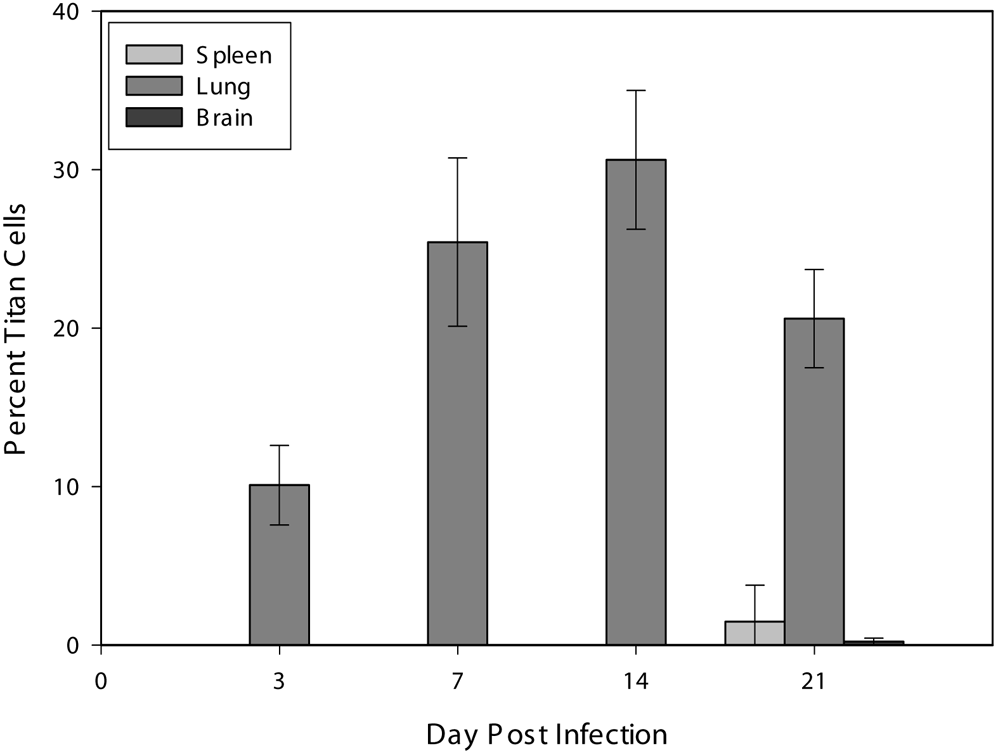

Supplement: Figure S1 — Titan cell formation in the lungs, spleen, and brain. Mice were intranasally infected with 5×10^4^ a or α cryptococcal cells. Lungs, brain, and spleen were collected at 1, 3, 7, 14 or 21 dpi. Samples were fixed in 10% formalin and stained with hematoxylin and eosin (H&E). The percentage of titan cells (>10 µm in diameter) was determined by microscopic examination of >500 cells per sample per mouse. Sufficient cell numbers were unavailable in tissue sections from 1 dpi lungs and 1, 3, 7, and 14 dpi spleen and brain for quantification. Error bars indicate SD from six mice per time point. (2.29 MB TIF) [file ppat.1000953.s001.tif]

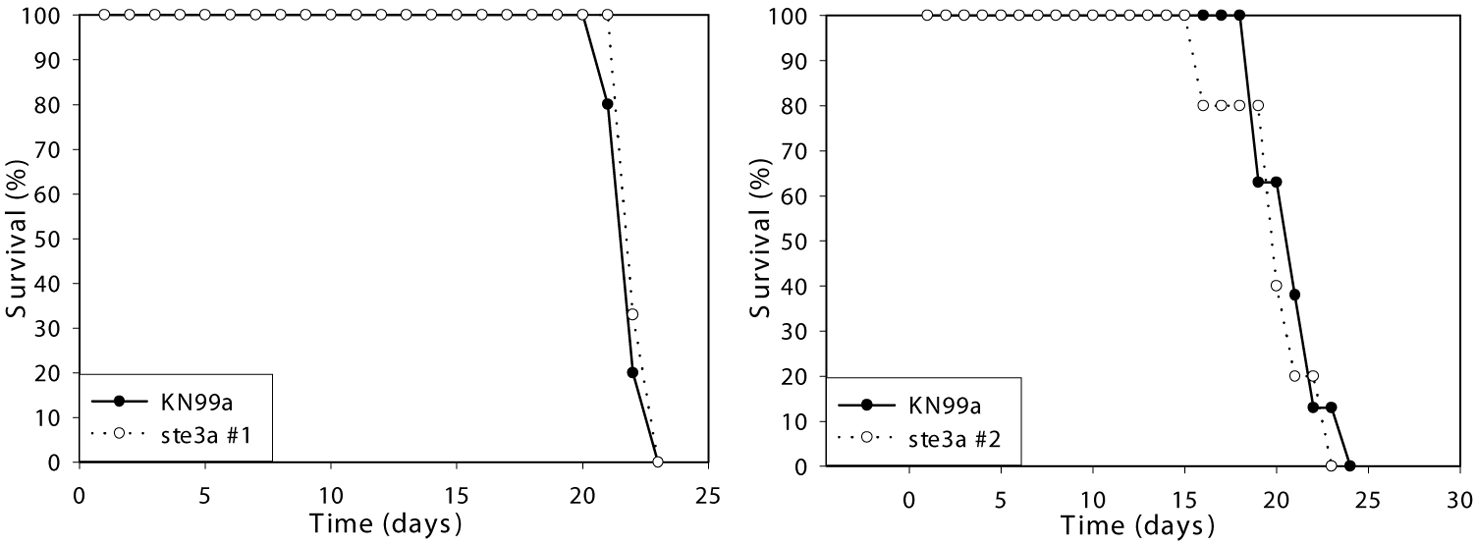

Supplement: Figure S2 — ste3aΔ survival assays. Mice were inoculated with 5×10^4^ cells of either wild-type a, ste3aΔ#1 (left) or ste3aΔ#2 (right) cells and progression to morbidity was monitored. (2.43 MB TIF) [file ppat.1000953.s002.tif]

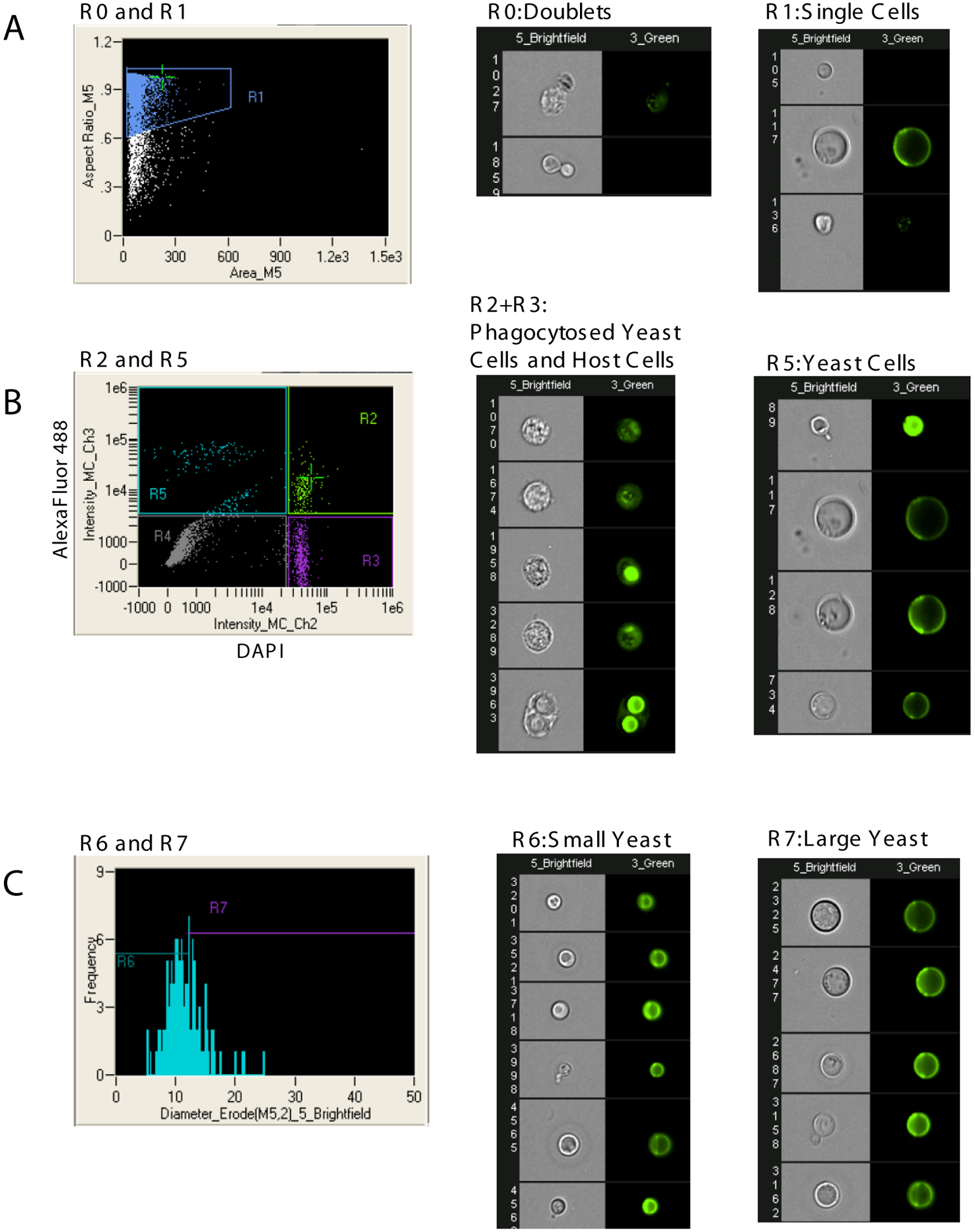

Supplement: Figure S3 — Imaging Flow Cytometry. C. neoformans a and α strains were combined with AlexaFluor 488 (green) and incubated at 25°C for 20 minutes. Cells were washed with sterile PBS to remove excess dye. Mice were inoculated with an approximate 1∶1 ratio of a:α cells. At 3 days post-infection animals were sacrificed and BALs were performed. The resulting cells were fixed, DAPI stained, and analyzed using an ImageStream flow cytometer using IDEAS software (Amnis Corporation). A) Cells were first examined for single cells (R1). Aggregates and doublets were excluded from further analysis. B) The R1 population was analyzed for DAPI intensity (X-axis) and AlexaFluor 488 intensity (Y-axis). DAPIhi host cells and phagocytosed cryptococcal cells (R2 and R3) as well as unstained yeast cells (R4) were excluded from further analysis. C) Diameter was used to divide the remaining population, R5, into cells <10 µm (R6) and >10 µm (R7). Samples from four mice per treatment were analyzed and gates determined by consensus among the samples. (8.29 MB TIF) [file ppat.1000953.s003.tif]

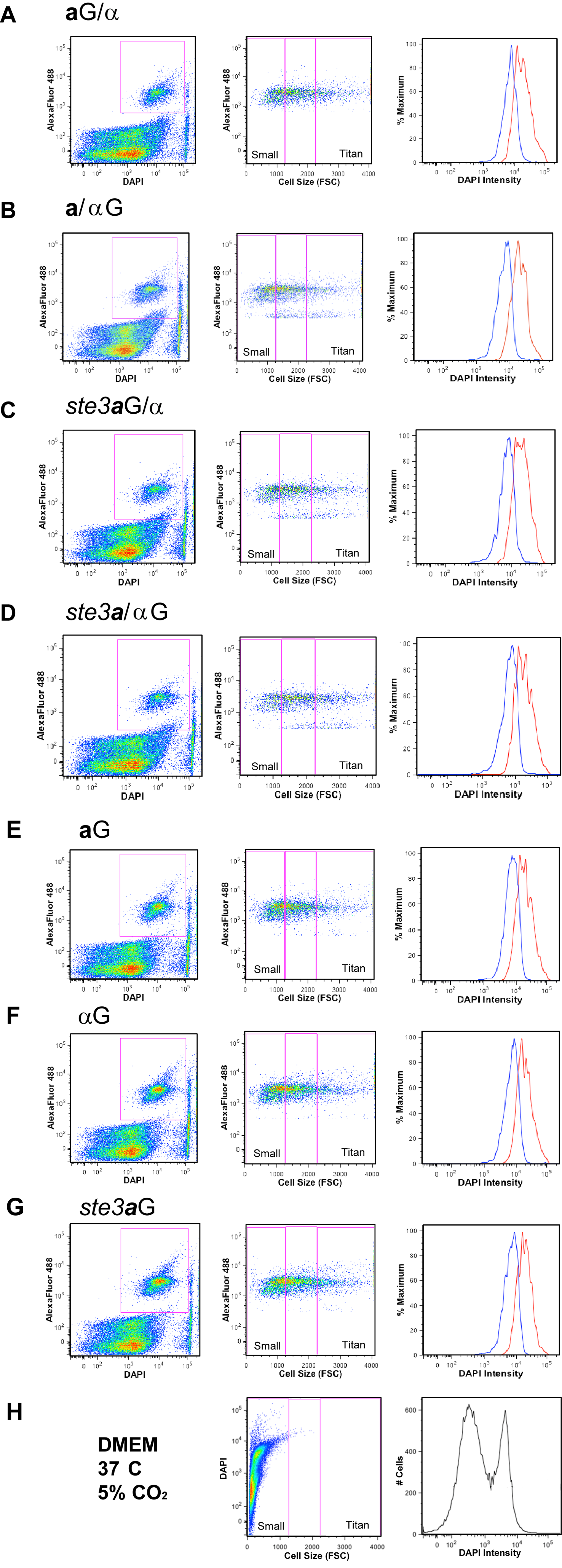

Supplement: Figure S4 — C. neoformans titan cells are polyploid. a and α strains were combined with AlexaFluor 488 (green) and incubated at 25°C for 20 minutes. Cells were washed with sterile PBS to remove excess dye. Mice were inoculated with an approximate 1∶1 ratio of a:α cells. At 3 days post-infection animals were sacrificed and BALs were performed. The resulting cells were fixed, DAPI stained and analyzed using an LSRII flow cytometer using FACSDiva software (BD). Fluorescently labeled yeast cells were first identified as 488hi and DAPIlow (left). Forward scatter (FSC) was used to identify small (≤10 µm) and titan (>20 µm) cells. Small (blue line) and titan (red line) cell populations were analyzed for DNA content (DAPI) and normalized for cell number (right). A–D coinfections E–G individual infections H) C. neoformans cells were grown for 5 days at 37°C and 5% CO2 in DMEM, fixed and DAPI stained. Both 1C and 2C peaks can be seen in this cell population. Absolute levels of DAPI intensity in these control cells varied from experiment to experiment thus were included as internal controls for every experiment. (2.31 MB TIF) [file ppat.1000953.s004.tif]

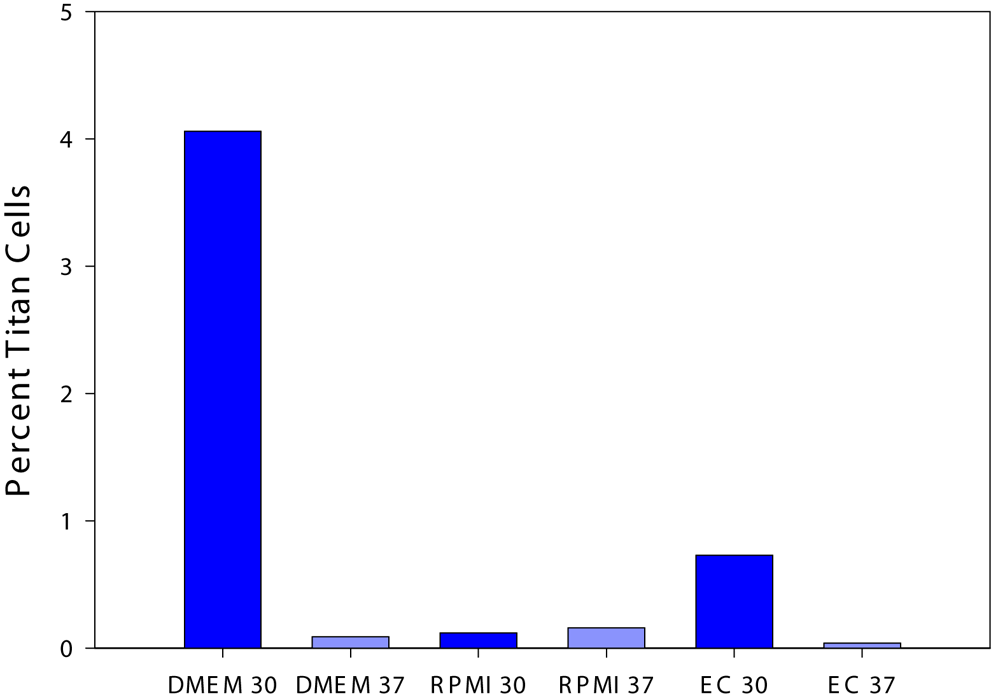

Supplement: Figure S5 — In vitro titan cell production. Cryptococcal cells were grown in spent DMEM (MH-S alveolar macrophages), RPMI (MH-S alveolar macrophages), or endothelial cell media (human umbilical vein endothelial cells, HUVEC) at 30°C or 37°C. Samples were fixed in 3.7% formaldehyde and 50,000 cells per sample were analyzed for cell size (forward scatter). Data presented are representative of five independent experiments. (2.12 MB TIF) [file ppat.1000953.s005.tif]
